# Quality of life assessment and its spatial correlation in impoverished districts and counties: A case study of Guizhou Province

**DOI:** 10.3389/fpubh.2023.1153953

**Published:** 2023-03-27

**Authors:** Junyue Yang, Jia Du, Heng Yang, Canhui Cheng, Tingting Chen

**Affiliations:** ^1^College of Architecture and Urban Planning, Guizhou University, Guiyang, China; ^2^School of Architecture and Urban Planning, Chongqing University, Chongqing, China; ^3^Guizhou Detection Technology Research and Application Center, Guiyang, China

**Keywords:** quality of life assessment, GIS, spatial correlation, impoverished counties, Guizhou Province

## Abstract

China’s rapid urbanization has greatly boosted the quality of life of its traditionally impoverished regions. Research into the spatial distribution characteristics, evolution and spatial correlation of the quality of life in impoverished regions can help illuminate the experience of successful development and construct a knowledge base for authorities to devise development strategies. This study focuses its attention on the historically impoverished districts and counties (which are designated as parallel administrative units in China) of Guizhou Province in southwestern China. Extensively citing official statistics on districts and counties released by China’s National Bureau of Statistics and local governments, it assesses the quality of life of those places in the 3 years of 2000, 2010, and 2020 from the four dimensions of economy, society, culture, and environment. The aim is to illustrate the distribution characteristics and the evolution of quality of life in Guizhou’s historically impoverished districts and counties. In order to understand the characteristics of spatial clustering as well as the patterns of evolution of the quality of life of Guizhou’s impoverished districts and counties, the study incorporates spatial autocorrelation analysis into a spatio-temporal analysis of local quality of life. It could presumably help enrich the knowledge base that local authorities draw on to formulate development strategies that are scientific and adapted to local conditions. The study found that while the overall quality of life in all the impoverished districts and counties of Guizhou Province has improved, large gaps in quality of life between eastern and western regions of the province persisted. In addition, the driving force behind the evolution in the overall quality of life of those places changed with time, as did the characteristics of the spatial aggregation in quality of life.

## 1. Introduction

While often casually used to describe how good life is, the concept of quality of life in technical and scholarly contexts often covers a wide range of aspects. Its definitions are numerous and vastly different (1), but improving the quality of life is always the desired outcome of urban development. Defining quality of life must be based on specific contexts, so as to ensure the coherence between the definition of the concept and the execution of the research (2). Among existing quality of life (QoL) research projects, most adopt the WHO’s definition of the concept, which views quality of life as “an individual’s perception of their position in life in the context of the culture and value systems in which they live and in relation to their goals, expectations, standards and concerns” (3). It is generally believed that quality of life is a comprehensive measurement of the physical, mental, social well-being of an individual or of a group. Notably, a large number of studies have demonstrated that the natural environment in which people live is of great importance to their physical and mental health (4). Their financial status also has a crucial impact on their physical, mental and social existence (5). In addition, cultural activities and cultural atmosphere also play a role in promoting people’s quality of life (6). Taking all relevant factors into consideration, this study defines quality of life as the sense of satisfaction felt by the individual or the group from their physical well-being and economic, cultural, social and environmental states. It approaches QoL assessment mainly from the angle of urban–rural development.

Research on quality of life has always been considered of great referential value for decision-making, particularly when it takes sustainable development into consideration (7, 8). QoL assessment has been widely used in urban research. It can yield valuable information pertaining to human well-being, urban environmental quality, etc., and influence social progress by shaping relevant policies (9). It can also help elucidate the development experience of a specific places and facilitate the understanding of interregional differences and interactions (10). In addition, by investigating the specific dimensions constituting the overall QoL, urban QoL assessment can also shed light on factors driving the evolution of quality of life (11).

Many scholars have discussed the dimensions of urban QoL. Such discussions are broadly oriented toward three perspectives—personal development, urban development, and a comprehensive perspective combining the two.

An example focusing on the first perspective is that of Robert, who defines quality of life as a combination of the individual’s physical health and mental health, both indispensable to personal development, satisfaction, happiness, and well-being (8). Shafer et al. propose that urban QoL encompasses the four dimensions of mobility, accessibility, conviviality and sustainability (12). Smith et al. dissect urban QoL by considering six aspects—livability, diversity, personal freedom, character, mobility and connection (13). Broadly speaking, urban QoL researches emphasizing personal development come mainly from medical science or social sciences and they tend to pay special attention to the quality of life of disadvantaged groups (14).

Adopting the perspective of urban development, Marans in his urban QoL research believes that urban QoL is a composite concept consisting of multiple dimensions such as society, economy, culture, politics, environment and psychology, and that it is generally used to describe people’s living conditions (15). Morais et al. divide urban QoL into nine dimensions, namely society, economy, environment, population, public participation, education and training, culture and entertainment, information society, transportation and tourism (16). Węziak-Białowolska argues that urban QoL is constituted by social, economic, institutional, environmental, and physical factors (17).

Urban QoL researches considering both personal development and urban development include that of ANA et al., which highlights the psychological dimension in urban QoL assessment, integrates it into the perspective of urban development, and proposes five dimensions to be considered in urban QoL assessment—psychological, social, economic, political, physical, environmental and mobility-oriented (18). Feneri et al. highlight the subjective feelings of individuals in QoL assessment, and combining them with urban development, propose six broad considerations in urban QoL assessment - environment, infrastructure, safety, health, transportation and others (19).

It is worth noting that, as a composite and interactive system, cities have both the character of a human society and that of a natural ecology. The former is reflected in the activities in and models of economy, culture, and social security, etc.; the latter is based on healthy ecological processes and complete ecosystem services (20). An analytical perspective factoring in both human society and natural ecology can help promote a more thorough understanding of an urban QoL guided by the idea of sustainability (21). Based on a survey conducted in 51 European cities, the research of Vincenzo et al. investigates the impact of green space on the quality of life and points out that urban green space can significantly boost the quality of life (22). In human society, culture often provides the core brand value of a city. It runs through every segment of the city’s operation. The culture of a city is increasingly functioning as a catalyst for the fusion of quality-of-life improvement and urban economic growth. Therefore, the cultural dimension is very important in urban QoL research (23).

Most of the existing studies on urban QoL assessment have served as tools for conducting rankings and making guides (24), and Providing guidance on making development policies. Naturally, their attention is primarily on cities: some deal with the QoL of a single city (14, 18, 25); some compare that of multiple cities (16, 26, 27); some draw comparisons between different areas within a city (28–30), and still some compare cities within a country (31). However, in recent years, there has been a growing trend toward researching the QoL assessment of counties as an administrative unit. For instance, Ma et al. analyzed the spatial patterns of the degrees of urban–rural integration in China’s Gansu Province by assessing the quality of life in 87 counties in the province. Their research covers three QoL dimensions—economic, social and environmental (32).

Purely quantitative methods or hybrid methods combining qualitative and quantitative analysis have been used in urban QoL assessments. Those primarily oriented toward personal development mostly employed hybrid methods (18, 33), the most common of which is questionnaire survey (14, 19, 25, 27, 30). Those focusing on urban development mostly used quantitative methods, in which statistical analysis methods and tools such as importance performance analysis (IPA), information entropy, equalization index, structural equation modeling, and hierarchical multiple linear regression analysis have been widely applied (11, 32, 34). Another widely used tool is GIS, which has grown into an important work platform in urban QoL assessments involving spatial pattern analysis (28, 35). Also playing a crucial role in such assessments is spatial autocorrelation analysis (32, 35).

Now a popular tool in urban research (36), spatial autocorrelation analysis allows for the analysis of the dynamic evolution of urban spatial relationships under spatiotemporal sequences (37, 38), and can illuminate the characteristics of the region by analyzing the characteristics of surrounding areas. It provides a new perspective for the analysis of urban spatial characteristics (39, 40). At present, spatial autocorrelation analysis in urban research is mainly applied in the understanding of topics related to urban environmental change, such as spatiotemporal change patterns of urban heat islands (41), spatial characteristics of urban atmospheric environmental efficiency (42, 43), spatial characteristics of urban ecosystem services (44), and urban and rural animal habitats. (45). More notably, the usefulness of spatial autocorrelation analysis in urban management research has also received recognition. Kim et al. used spatial autocorrelation analysis to interpret the regional spatial relationship of smart city services in South Korea (46). Balducci et al. used it to interpret the spatial pattern of the intelligent management of major towns in Italy (47). Li et al. used both global and local spatial autocorrelation analysis to investigate the spatial dynamic characteristics of regional economic disparity among cities in the Yangtze River Delta (40). Huang et al. interpreted the degree of spatial autocorrelation of housing transaction volumes in the counties and cities of Taiwan (48). Li and Derudder tried to use the spatial autocorrelation model to identify the population centers and sub-centers of Chinese cities and dissect the mechanism of the evolution of urban population centers (49). A study particular of note is that of Faka et al., who applied spatial autocorrelation analysis to urban QoL assessment at the local level to illuminate the characteristics of clustering in the urban QoL of Athens (35). The study provides new insights into how to analyze the spatial evolution of urban quality of life. The introduction of spatial autocorrelation analysis into QoL assessment is conducive to explaining the possible spatial correlation of quality of life changes. In other words, the characteristics of one region’s quality of life, along with its patterns of change and the driving forces behind the change can be understood by analyzing the characteristics of the QoL of surrounding regions. Thus, spatial autocorrelation analysis can enrich and improve the methodology for QoL analysis.

Concerned with the QoL evolution in poor regions, this urban development study evaluates the QoL in Guizhou’s impoverished districts and counties by means of quantitative analysis. The QoL assessment is realized through evaluating the four specific dimensions of economy, society, culture and environment. By comparing the quality of life of those places in the 3 years of 2000, 2010, and 2020, this study seeks to elucidate the distribution characteristics and evolution patterns of the quality of life in Guizhou’s impoverished districts and counties. Through introducing spatial autocorrelation analysis into the spatio-temporal analysis of regional urban QoL, it sheds light on the possible spatial clustering of the quality of life of Guizhou’s impoverished districts and counties and its change patterns. As such, it can help local authorities better understand their experience of development and make better policies to improve people’s quality of life.

## 2. Data processing

### 2.1. Scope of the study

This study evolves around the mountainous Guizhou Province in southwestern China. Guizhou has long been designated as an economically backward province in China. Of its 88 districts and counties, 66 were on China’s poverty list, making it the province with the largest poor population in the country. However, by November 2020, all 66 places had been successfully removed from the list, making Guizhou the province with the largest number of people lifted out of poverty in China. From the province with the largest impoverished population to the one with the largest population lifted from poverty, this change of Guizhou’s status in China has undoubtedly been accompanied by a dramatic transformation in the life in Guizhou’s historically impoverished districts and counties. This can be attested by the fact that the annual GDP of the province increased from 0.10 trillion yuan in 2000 to 1.79 trillion yuan in 2020 and its area of construction land expanded by 2,493 square kilometers over the same period. By analyzing the distribution characteristics and change patterns of the quality of life of Guizhou’s impoverished districts and counties, the study can sum up the experience of successful development of those places, which may both serve the needs of Guizhou in consolidating its progress and be drawn on by other places around the globe when combating poverty. Thus, taking the 66 historically impoverished districts and counties of Guizhou Province as its subjects, this study attempts to analyze the evolution of the quality of life of those places in the two decades from 2000 to 2020, and explore the spatial correlations between different places in the evolution of their quality of life.

### 2.2. Data sources

The data used in this study mainly comes from China Statistical Yearbook (County-Level), which offers a comprehensive look into the socio-economic development of China’s counties (and districts as an equivalent administrative unit). Additional data sources include *Guizhou Statistical Yearbook*, *China Energy Statistical Yearbook*, “Globeland30” (a global land cover (GLC) data product from the website GLOBALLANDCOVER), and the lists of Guizhou’s national and provincial intangible cultural heritage items and protected cultural relics, which are published on the official website of the people’s government of Guizhou Province.

### 2.3. Data processing (standardization)

Given the difference in the dimensions of various data, the research adopts the normalization method to render the data dimensionless. The formula for calculating positive indicators is as follows:


$$X\prime_{i}=\frac{X_{i}-X_{imin}}{X_{imax}-X_{imin}}$$

The formula for calculating negative indicators is as follows:


$$X\prime_{i}=\frac{X_{\mathrm{imax}}-X_{i}}{X_{\mathrm{imax}}-X_{\mathrm{imin}}}$$

In the formula: $\;X_{i}$ stands for the original statistic of category i in the index system of the QoL assessment of the districts and counties of Guizhou Province; ${\mathrm{iX}}_{\mathrm{imax}}$ stands for the maximum value of the original statistics of category i in the index system of the QoL assessment; $X_{\mathrm{imin}}$ stands for the minimum value of the original statistics of category i in the index system of the QoL assessment; $\mathrm{iX}\prime_{i}$ stands for the utility value of category i in the index system of the QoL assessment.

## 3. Research design and methodology

### 3.1. Quality of life assessment index system construction and weights setting

As is widely known, the economy, culture, society, and ecology of cities and towns together form an internally unified and organic whole. As such, urban QoL assessment needs to be approached with systematic thinking. To fully represent the levels of economic development, cultural prosperity, social stability and ecological health of a given place, this study constructed a QoL assessment index system comprising four secondary indicators, namely economic QoL index, cultural QoL index, social QoL index and environmental QoL index.

All the metrics and data cited in this study were obtained from the database for counties established by China’s National Bureau of Statistics, the publicly accessible information released by the People’s Government of Guizhou Province and the land cover data provided by GlobeLand30.

Among the four dimensions of quality of life measured in this study, economy provides the material foundation of a high quality life. The economic QoL must take into consideration both micro-factors such as income, savings and consumption, as well as the state of economic development as a macro-factor. As such, this study devised three third-level indicators of economic QoL, including income index, consumption index and vitality of industries index. The income index is calculated by considering the annual GDP of a place and the total savings of its residents. The consumption index is calculated by considering the amount of electricity consumption and the number of telephone subscribers at year-end. The vitality of industries index is calculated by factoring in the added values of the primary, secondary and tertiary sectors of the economy, the area occupied by facility agriculture and the number of industrial enterprises above designated size. Altogether, the three third-level indicators of economic QoL are represented by nine fourth-level ones.

Another QoL dimension examined in this study is culture, which forms the intellectual and spiritual foundation for a high-quality life. The study evaluates the cultural QoL of Guizhou impoverished districts and counties by considering three elements: the capacity of cultural transmission, the number of material cultural items and that of non-material cultural items. The capacity of cultural transmission is reflected in the number of students presently enrolled in schools. The numbers of material and non-material cultural items are represented by the numbers of tangible and intangible cultural heritage items respectively, which are designated by China’s national cultural authority or its provincial divisions. In total, the cultural QoL of Guizhou’s impoverished districts and counties are reflected in five fourth-level indicators.

A third QoL dimension evaluated in this study is social conditions, which are also indispensable when measuring quality of life. The study approaches social QoL from the three perspectives of employment, social welfare and medical services. The state of employment is examined by looking at the proportions of people employed in the secondary and tertiary sectors of the economy. Social welfare is reflected in the number of social welfare institutions as well as the number of beds provided by them. Medical services are assessed through counting the number of beds offered in health care facilities. All in all, the social QoL comprises four fourth-level indicators.

The environmental QoL index in this study considers not only the natural resources of a place, but also the level of environmental pollution it suffers. Altogether, this index is represented by three third-level indicators, namely green space resources, blue space resources and carbon emissions, which correspond with three fourth-level indicators. It is worth noting that carbon emissions are a reverse indicator (see Table 1). Besides, the study utilizes the AHP-Analysis Hierarchy Process to set the weights of the indicators.

**Table 1:** QoL assessment index system and weights setting.

QoL dimensions	Indicators	Explanation
Economic QoL index (0.12)	Income index (0.35)	Annual GDP (10,000 yuan) (0.49)
		Balance of savings at year end (10,000 yuan) (0.51)
	Consumption Index (0.32)	Electricity consumption (kWh) (0.47)
		Number of telephone subscribers at year end (household) (0.53)
	Vitality of industries (0.33)	Added value of the primary sector of the economy (10,000 yuan) (0.25)
		Added value of the secondary sector (10,000 yuan) (0.16)
		Added value of the tertiary sector (10,000 yuan) (0.23)
		Area covered by facility agriculture (hectares) (0.06)
		Number of industrial enterprises above designated size (0.30)
Cultural QoL index (0.13)	Education (0.34)	Number of students enrolled in middle schools (person) (1.00)
	Intangible culture (0.45)	Number of national intangible cultural heritage items (0.24)
		Number of provincial intangible cultural heritage items (0.76)
	Material culture (0.21)	Number of major historical and cultural sites protected at the national level (0.45)
		Number of major historical and cultural sites protected at the provincial level (0.55)
Social QoL index (0.16)	Employment (0.35)	The proportions of residents employed in the secondary and tertiary sectors (1.00)
	Social welfare (0.40)	Number of social welfare institutions (0.72)
		Number of beds in social welfare institutions (0.28)
	Medical services (0.25)	Number of beds in health care facilities (1.00)
Environmental quality of life index (0.59)	Green space resources (0.47)	Area of cultivated land and land covered by forests, grass, or shrubs (hectares) (1.00)
	Blue space resources (0.01)	Area of waters and wetlands (hectare) (1.00)
	Carbon emissions* (0.52)	Annual carbon emissions (million metric tons) (1.00)*

*Indicates a reverse index, the weights are in parentheses.

### 3.2. Correlation analysis

The study uses Pearson Correlation Analysis to explain the correlations between the overall quality of life and the levels of economic development, cultural prosperity, social stability and ecological health in Guizhou’s districts and counties across different periods. The Pearson correlation coefficient, also known as the product-difference correlation coefficient, is a statistical indicator that depicts the degree and direction of the linear correlation between two variables. The correlation coefficient is a dimensionless statistical indicator, and its value ranges from −1 to 1. When below zero, it indicates a negative correlation, and when above 0, it indicates a positive correlation. A zero means there is no correlation. The larger the absolute value of the correlation coefficient, the higher the correlation between the two variables.

### 3.3. Spatial autocorrelation analysis

Spatial autocorrelation analysis is classified into global autocorrelation analysis (also called Global Moran’s I) and local autocorrelation analysis (or Local Moran’s *I*). Both types of spatial autocorrelation analysis can be performed in the software ArcGIS. Through Global Moran’s I, this study firstly determined whether the quality of life in Guizhou’s districts and counties had a spatial autocorrelation, that is, whether places with high quality of life were surrounded by those of the same kind. If there was a global spatial autocorrelation in the quality of life, then the study would proceed to calculate the Local Moran’s *I* to find out where of those places showed aggregations or were outliers in terms of quality of life.

#### 3.3.1. Global Moran’s *I* analysis

The study first performed a global spatial autocorrelation analysis on the QoL assessment results of Guizhou’s districts and counties in 2000, 2010, and 2020. This was meant to determine whether there were spatial aggregations in the quality of life of those places. The global spatial autocorrelation analysis covered the overall QoL and the QoL as reflected in four specific dimensions—economy, culture, society, and environment.

The Global Moran’s *I* was developed by Australian statistician Patrick Alfred Pierce Moran in 1948 (50). Based on the autocorrelation between the position of the elements and the attribute value in a global space, this index may classify global autocorrelation modes into three types—cluster mode, discrete mode, or random mode. Global autocorrelation analysis mainly evaluates the significance of autocorrelation by calculating the Moran’s *I* value, the *z*-score and the value of *p* (probability).

Its calculation formula is as follows:


$$I=\frac{n}{S_{0}}\;\frac{\sum_{i=1}^{n}\sum_{j=1}^{n}W_{i,j}Z_{i}Z_{j}}{\sum_{i=1}^{n}Z_{i}^{2}}$$

In it, $\;Z_{i}$ is the deviation between the attribute of element i and the mean value ( $x_{i}$ - $\bar{X}$ ); $W_{i,j}$ is the spatial weight between elements i and j ; n equals the total number of elements; and $S_{0}$ is the aggregation of all spatial weights.


$$S_{0}=\overset{n}{\underset{i=0}{\sum }}\overset{n}{\underset{j=1}{\sum }}W_{i,j}$$

The z-score is calculated through the following formula:


$$Z_{I}=\frac{I-E\left[I\right]}{\sqrt{V\left[I\right]}}$$

In it:


E[I]=−1/(n−1)

$$V\left[I\right]=E\left[I^{2}\right]-E{\left[I\right]}^{2}$$

Typically, the Global Moran’s *I* is between −1 and 1. If the Moran’s *I* is larger than zero, it indicates a positive spatial correlation, and the larger the value, the stronger the correlation. A Moran’s *I* below zero suggests a negative spatial correlation. The smaller the value, the greater the spatial difference. If the Moran’s *I* is zero, then the spatial correlation is random.

The value of *p* indicates probability. For pattern analysis tools, the value of *p* represents the probability that the observed spatial pattern is created through a random process. A small value of *p* suggests that the observed spatial pattern is unlikely to be generated through random processes (in other words, such was a small probability event).

The *z*-score represents the deviation from the standard. The larger its absolute value, the higher the degree of clustering. A *z*-score close to zero indicates that no significant clustering exists within the studied area. A positive *z*-score indicates a cluster of high values. A negative *z*-score indicates a cluster of low values. Therefore, by analyzing the *z*-score and value of *p* yielded by the calculation tool in ArcGIS, the confidence degree of the spatial autocorrelation of the calculation results can be determined (see Table 2).

**Table 2:** Global spatial autocorrelation confidence degree analysis.

*z*-score	Value of *p*	Confidence degree
< −1.65 or >+1.65	<0.10	90%
< −1.96 or >+1.96	<0.05	95%
< −2.58 or >+2.58	<0.01	99%

#### 3.3.2. Local Moran’s *I* analysis

By calculating the Global Moran’s *I*, the overall characteristics of aggregation in the spatial distribution of the quality of life-related indicators of Guizhou Province can be known, but these overall characteristics cannot show the specific clustering locations of the indicators in the space. Therefore, the study needed to present the spatial distribution of the clusters of the QoL indexes of Guizhou’s districts and counties by calculating the Local Moran’s *I*.

The Local Moran’s I was proposed by professor Luc Anselin at Arizona State University in 1995 (51). The formula for calculating this index for each element is as follows:


$$I_{i}=\frac{x_{i}-\bar{X}}{S_{i}^{2}}\overset{n}{\underset{j=1,j\neq i}{\sum }}w_{\mathrm{ij}}\left(x_{j}-\bar{X}\right)$$

In it, *x*_i_ is an attribute of element i; X̅ is the mean value of the corresponding attribute; and w_ij_ is the spatial weight matrix between elements *i* and *j*.


$$S_{i}^{2}=\frac{\sum_{j=1,j\neq i}^{n}{\left(x_{j}-\bar{X}\right)}^{2}}{n-1}$$

Value *n* stands for the total number of elements.

After calculating the Local Moran’s *I*, this study located the hot spots (or high-value clusters) and cold spots (or low-value clusters) of the QoL indexes of Guizhou’s counties and districts in the 3 years on the maps. The data was then used for further comparative analysis. Here, high values mainly refer to outliers surrounded by low values, while low values mainly represent outliers surrounded by high values.

## 4. Research outcome

### 4.1. Analysis of the evolution of quality of life

#### 4.1.1. Analysis of the evolution of the overall QoL

**Figure 1:**
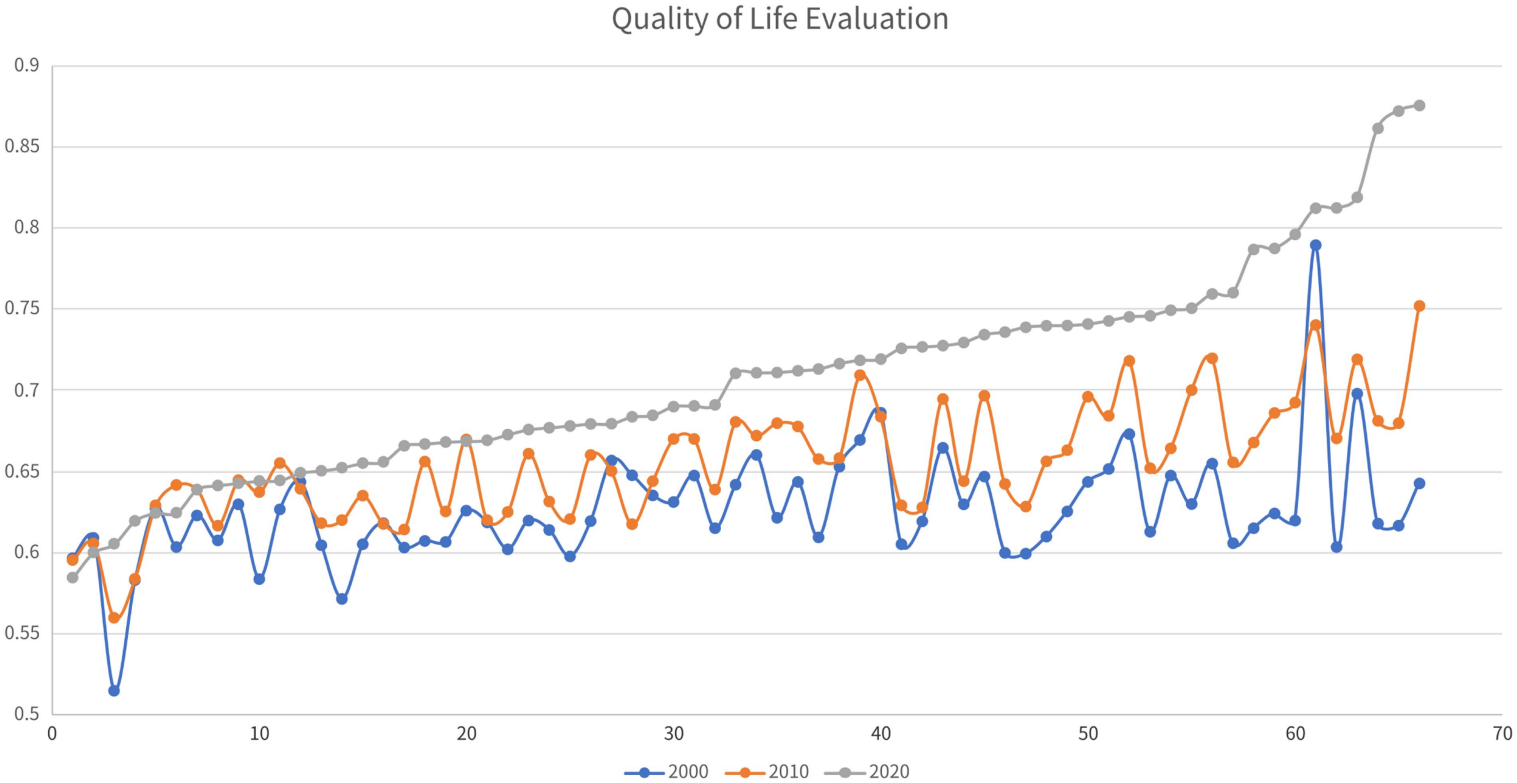
Analysis of the evolution of the quality of life of Guizhou’s districts and counties from 2000 to 2020.

As shown in Figure 1, in the two decades between 2000 and 2020, the overall quality of life in Guizhou’s impoverished districts and counties improved significantly. The places that have experienced the most significant QoL improvement are clustered in the southeast of the province. However, four districts and counties have seen their quality of life decline slightly over the two decades.

**Figure 2:**
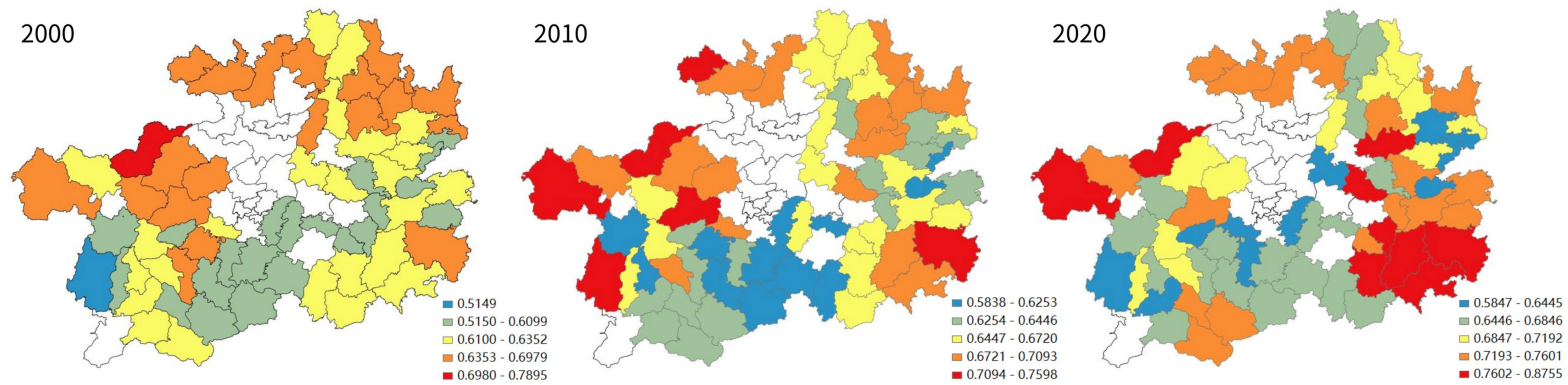
Spatial analysis of the changes in quality of life in Guizhou’s districts and counties.

From a spatial point of view (as shown in Figure 2) in 2000, among the impoverished districts and counties of Guizhou province, those with a high quality of life were clustered in the coal-rich northwest. In the decade between 2000 and 2010, the impoverished districts and counties where the quality of life rose the fastest were in Guizhou’s southeast, northwest, north and northeast. By 2010, the places with a high overall quality of life among Guizhou’s impoverished districts and counties had shifted to its southeast. A prominent example is Liping County, which achieved the most dramatic increase in its overall quality of life and came to be ranked No. 1 among Guizhou’s impoverished districts and counties in this regard. In the decade between 2010 and 2020, Guizhou’s impoverished districts and counties that saw the fastest improvement in quality of life were all in the southeast. In 2020, the vast majority of Guizhou’s historically impoverished districts and counties with the highest quality of life among peer places were in the southeastern part of the province. It can be seen that in the two decades between 2000 and 2020, the places with a higher quality of life among Guizhou’s historically impoverished districts and counties shifted from the northwest of the province to its southeast, which is rich in natural ecological landscapes and ethnic minority culture.

#### 4.1.2. Analysis of the evolution of the specific dimensions of quality of life

##### 4.1.2.1. Analysis of the evolution of the economic QoL

**Figure 3:**
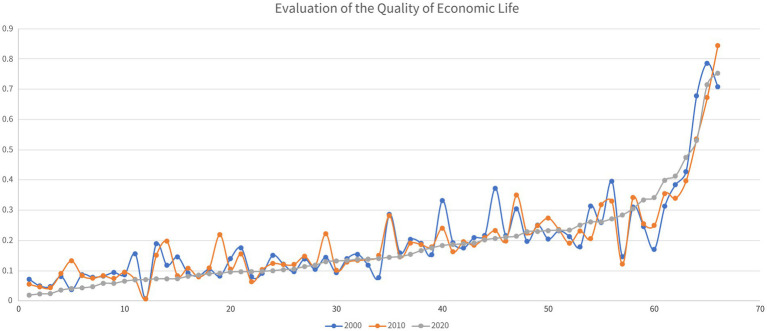
Analysis of the evolution of the economic QoL of Guizhou’s districts and counties from 2000 to 2020.

As shown in Figure 3, in the two decades from 2000 to 2020, the economic QoL of the 66 impoverished districts and counties of Guizhou province declined to varying degrees. In the first decade, the economic QoL of 37 of the 66 places worsened, and in the second decade, 43 places suffered declines in their economic QoL. Over the entire period, the overall economic QoL of 40 districts and counties declined.

**Figure 4:**
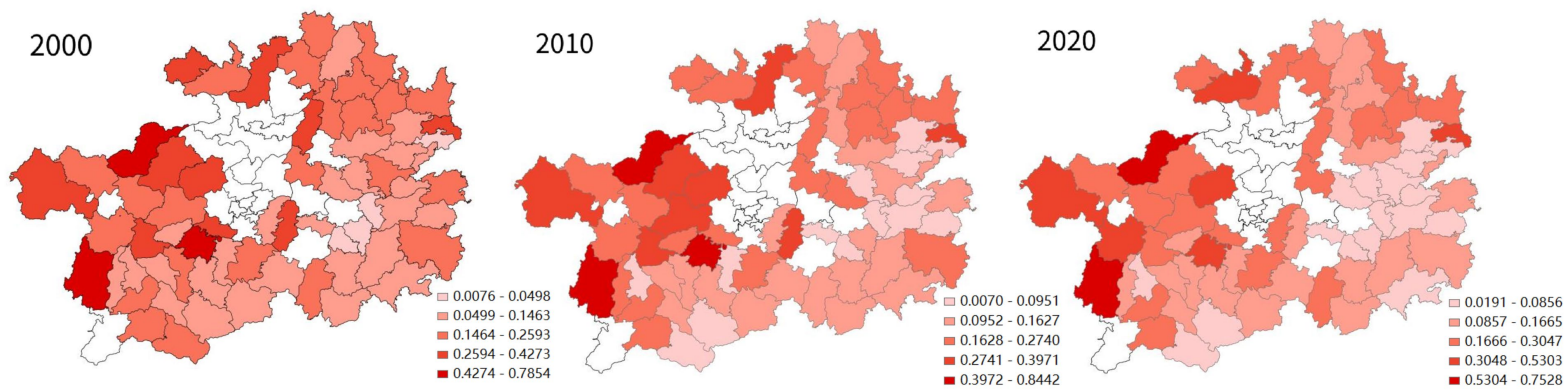
Spatial analysis of changes in the economic QoL of Guizhou’s districts and counties.

In terms of spatial distribution (see Figure 4), in 2000, the historically impoverished districts and counties of Guizhou Province with a high economic QoL were mainly located in its western, northern and eastern regions. In 2010 and 2020, the impoverished districts and counties with a relative high economic QoL remained concentrated in western and eastern Guizhou. In the decade from 2000 to 2010, the economic QoL of Guizhou’s impoverished districts and counties improved rapidly in the western and central regions of the province, whereas in the following decade ending in 2020, such an improvement happened not only to those two regions, but also to its north and east. Southern Guizhou remained an area with a low economic QoL.

##### 4.1.2.2. Analysis of the evolution of cultural QoL

**Figure 5:**
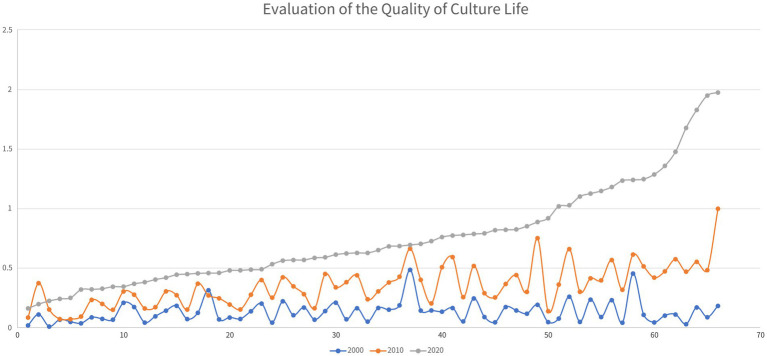
Analysis of the evolution of cultural QoL of Guizhou’s districts and counties from 2000 to 2020.

**Figure 6:**
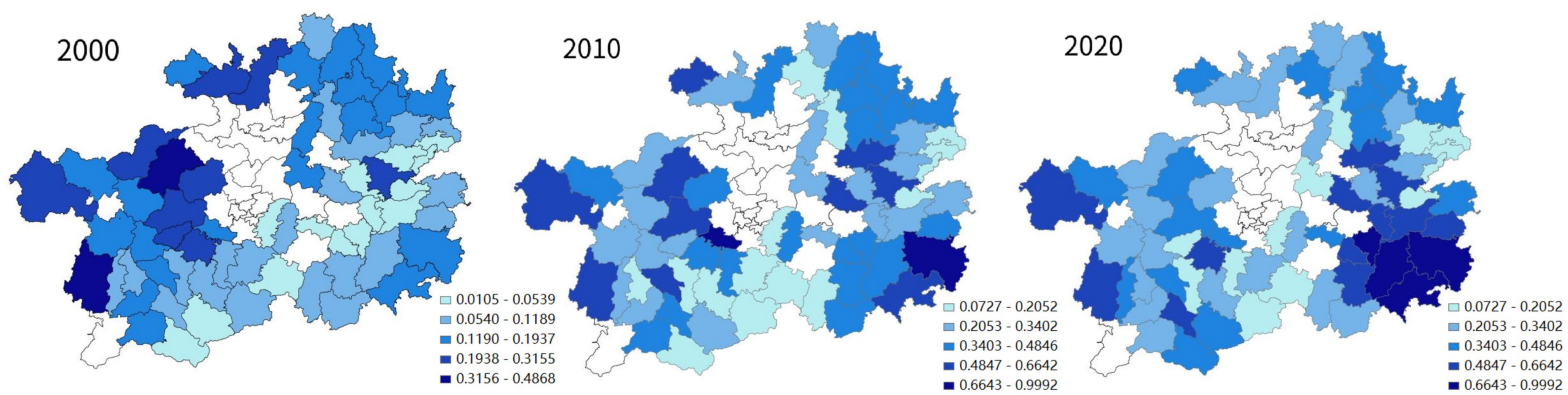
Spatial analysis of the changes in cultural QoL of Guizhou’s districts and counties.

In the two decades from 2000 to 2020, especially the second one, the cultural QoL in Guizhou’s impoverished districts and counties has increased by leaps and bounds (see Figure 5). At the same time, the spatial distribution of places with a relative high quality of cultural life undergone significant changes. As shown in Figure 6, in 2000, such places were all located in the western part of the province. In 2010, although Guizhou’s impoverished districts and counties with high quality of cultural life were still mostly in its western part, Liping County in the southeast topped this list. By 2020, the districts and counties with high cultural QoL in Guizhou had been completely relocated to the southeastern part of the province. Obviously, in the two decades, Guizhou’s impoverished districts and counties with high cultural QoL in Guizhou have been shifted from its west to its southeast.

##### 4.1.2.3. Analysis of the evolution of social QoL

**Figure 7:**
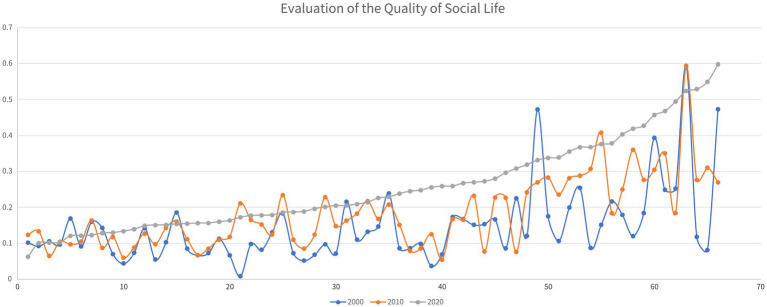
Analysis of the evolution of social QoL of Guizhou’s districts and counties from 2000 to 2020.

In the two decades between 2000 and 2020, the social QoL of the impoverished districts and counties of Guizhou Province has generally improved. In the decade between 2000 and 2010, 44 of the 66 impoverished districts and counties achieved improvement in their social QoL, while in the next decade, the social QoL of 55 impoverished districts and counties improved (see Figure 7).

**Figure 8:**
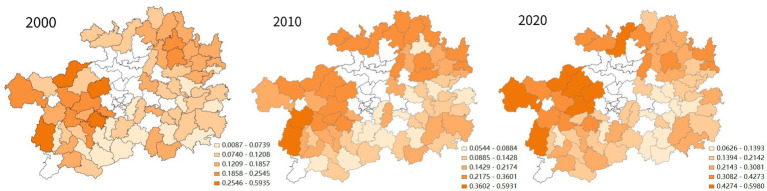
Spatial analysis of social QoL changes in districts and counties of Guizhou Province.

In terms of spatial distribution, no matter in 2000, 2010, or 2020, among Guizhou’s impoverished districts and counties, the ones with high social QoL were concentrated in the northwest of the province (see Figure 8). In the decade between 2000 and 2010, the impoverished districts and counties with rapid improvement in the quality of social life were in the west, north and east of Guizhou. In the next decade, the western or northern places in Guizhou saw the fastest improvement in their quality of social life.

##### 4.1.2.4. Analysis of the evolution of environmental QoL

**Figure 9:**
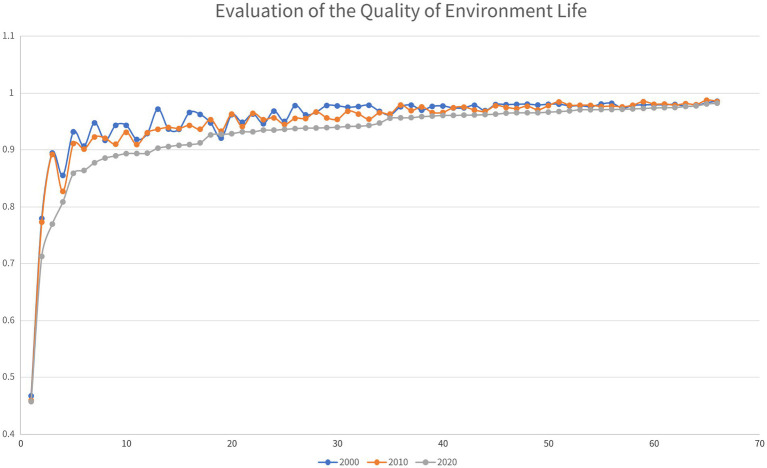
Analysis of the evolution of the environmental QoL of Guizhou’s districts and counties from 2000 to 2020.

The environmental QoL of Guizhou’s impoverished districts and counties generally (39 of all 66 places) went through slight declines during the decade from 2000 to 2010. In the next decade, all 66 impoverished districts and counties of Guizhou Province suffered notable declines in their environmental QoL (see Figure 9).

**Figure 10:**
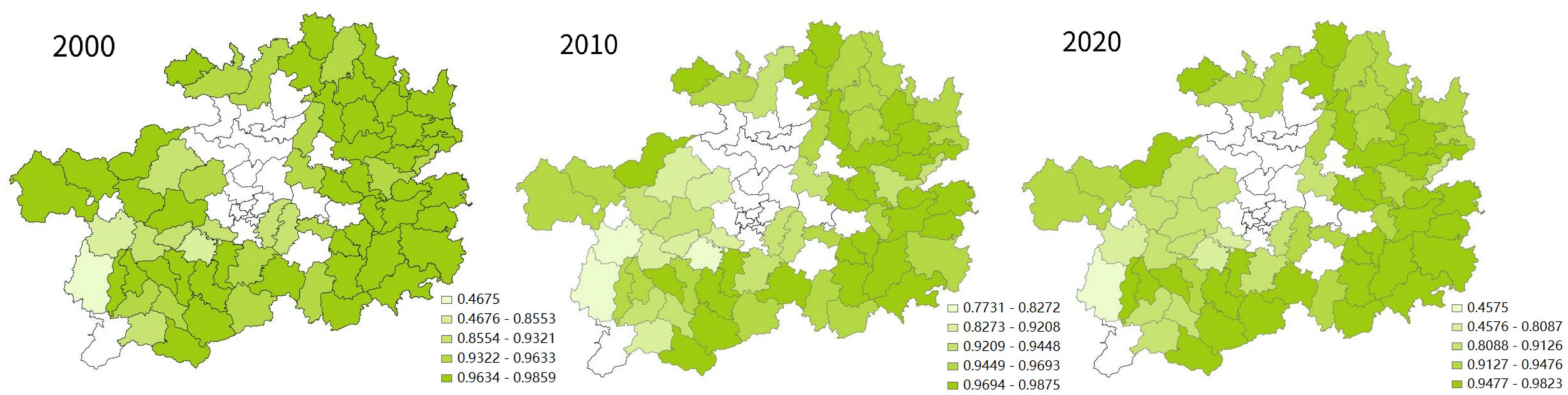
Spatial analysis of changes in the environmental QoL of Guizhou’s districts and counties.

Specifically, as shown in Figure 10, in 2000, the impoverished districts and counties in Guizhou with poor environmental QoL were all located in the southwest of the province; In 2010 and 2020, these places still had the worst environmental QoL. On the other hand, in 2000, Guizhou’s impoverished districts and counties with high environmental QoL were located in the southeast, east and northwest of the province. In 2010, the impoverished districts and counties with relatively high environmental QoL were located in the east, northwest and north of Guizhou; In 2020, the places that made the list were located in Guizhou’s east, south and north.

#### 4.1.3. Correlation analysis of the evolution of quality of life

As shown in Table 3, the Pearson correlation coefficient analysis was performed in the assessment of the overall QoL and the four secondary QoL indicators of Guizhou’s impoverished districts and counties in 2000, 2010, and 2020. It was found that there are huge differences in the correlations between the overall quality of life and the economic QoL, cultural QoL, social QoL and environmental QoL in the three different years.

**Table 3:** Analysis of Pearson correlation coefficient between the overall quality of life evaluations and the secondary indexes.

Year and index type	QoL assessment in 2000
Economic QoL in 2000	0.462**
Cultural QoL in 2000	0.373**
Social QoL in 2000	0.350**
Environmental QoL in 2000	0.394**
/	QoL assessment in 2010
Economic QoL in 2010	0.558**
Cultural QoL in 2010	0.739**
Social QoL in 2010	0.580**
Environmental QoL in 2010	0.022
/	QoL assessment in 2020
Economic QoL in 2020	−0.005
Cultural QoL in 2020	0.776**
Social QoL in 2020	0.212
Environmental QoL in 2020	0.381**

**p < 0.01.

In 2000, the overall quality of life in Guizhou’s impoverished districts and counties showed a clear positive correlation with their economic, social and environmental QoL, especially the first of the three. However, in 2010, while the overall quality of life of Guizhou’s impoverished districts and counties did not show a significant correlation with the local environmental QoL, its positive correlations with the economic QoL, cultural QoL and social QoL all increased significantly. Of the three specific QoL dimensions, its positive correlation with the cultural QoL was the most prominent. It is worth noting that in 2020, the overall quality of life of Guizhou’s impoverished districts and counties showed the strongest positive correlation with the local cultural QoL, followed by the environmental QoL, while it exhibited no significant correlation with their economic or social QoL. It is obvious that in different years, the secondary QoL indicators affecting the overall quality of life of Guizhou’s impoverished districts and counties were not the same, which implies that in the two decades, the development models of those places experienced constant evolution.

### 4.2. Quality of life spatial autocorrelation analysis

#### 4.2.1. Quality of life global spatial autocorrelation analysis

By calculating the Global Moran’s *I*s, *z*-scores and value of *p*s of the overall QoL, economic QoL, cultural QoL, social QoL and environmental QoL of Guizhou’s impoverished districts and counties in 2000, 2010 and 2020, we can determine whether there existed global spatial autocorrelation, or the phenomenon of spatial clustering, in different indexes. As shown in Table 4, in terms of overall QoL of Guizhou’s historically impoverished districts and counties, no matter in 2000, 2010 or 2020, there was consistently an obvious positive spatial correlation, meaning that high values were clustered together, as were low values, indicating a positive spatial correlation and thereby a conspicuous spatial clustering. However, the economic QoL indexes and social QoL indexes of Guizhou’s impoverished districts and counties did not exhibit obvious spatial clustering in 2000 (The underlined figures in Table 4 indicate that there was no obvious spatial clustering). Only in 2010 and 2020, there were clear spatial clustering in these indexes. Meanwhile, the cultural QoL indexes of Guizhou’s impoverished districts and counties exhibited spatial clustering in 2000 and 2010, which were, however, gone in 2020, while the environmental QoL indexes exhibited consistent positive spatial correlations across the 3 years.

**Table 4:** The Global Moran’s *I*s, *z*-scores, and value of *p*s of QoL across the years.

Year and index type	Moran *I*	z-score	*p*-value
Overall QoL in 2000	0.272379	3.772652	0.000162
Overall QoL in 2010	0.189075	2.236849	0.025296
Overall QoL in 2020	0.206635	2.801927	0.005080
Economic QoL in 2000	0.108392	1.591382	0.111524
Economic QoL in 2010	0.205945	2.893757	0.003807
Economic QoL in 2020	0.229392	3.129172	0.001753
Cultural QoL in 2000	0.339045	4.516181	0.000006
Cultural QoL in 2010	0.248641	3.308746	0.000937
Cultural QoL in 2020	0.051	0.0828129	0.407597
Social QoL in 2000	0.103887	1.544800	0.122395
Social QoL in 2010	0.357943	4.715855	0.000002
Social QoL in 2020	0.492622	6.256091	0.000000
Environmental QoL in 2000	0.111264	2.372154	0.017685
Environmental QoL in 2010	0.266849	3.700970	0.000215
Environmental QoL in 2020	0.189966	3.094898	0.001969

In other words, in the 3 years, the overall QoL and the environmental QoL in those places maintained positive spatial correlations, while the spatial clustering of the economic, social, and cultural QoL underwent changes. For the first two, the spatial clustering, once non-existent, came to exist later; and for the clustering of the cultural QoL, the change was a reverse one.

#### 4.2.2. Quality of life local spatial autocorrelation analysis

**Figure 11:**
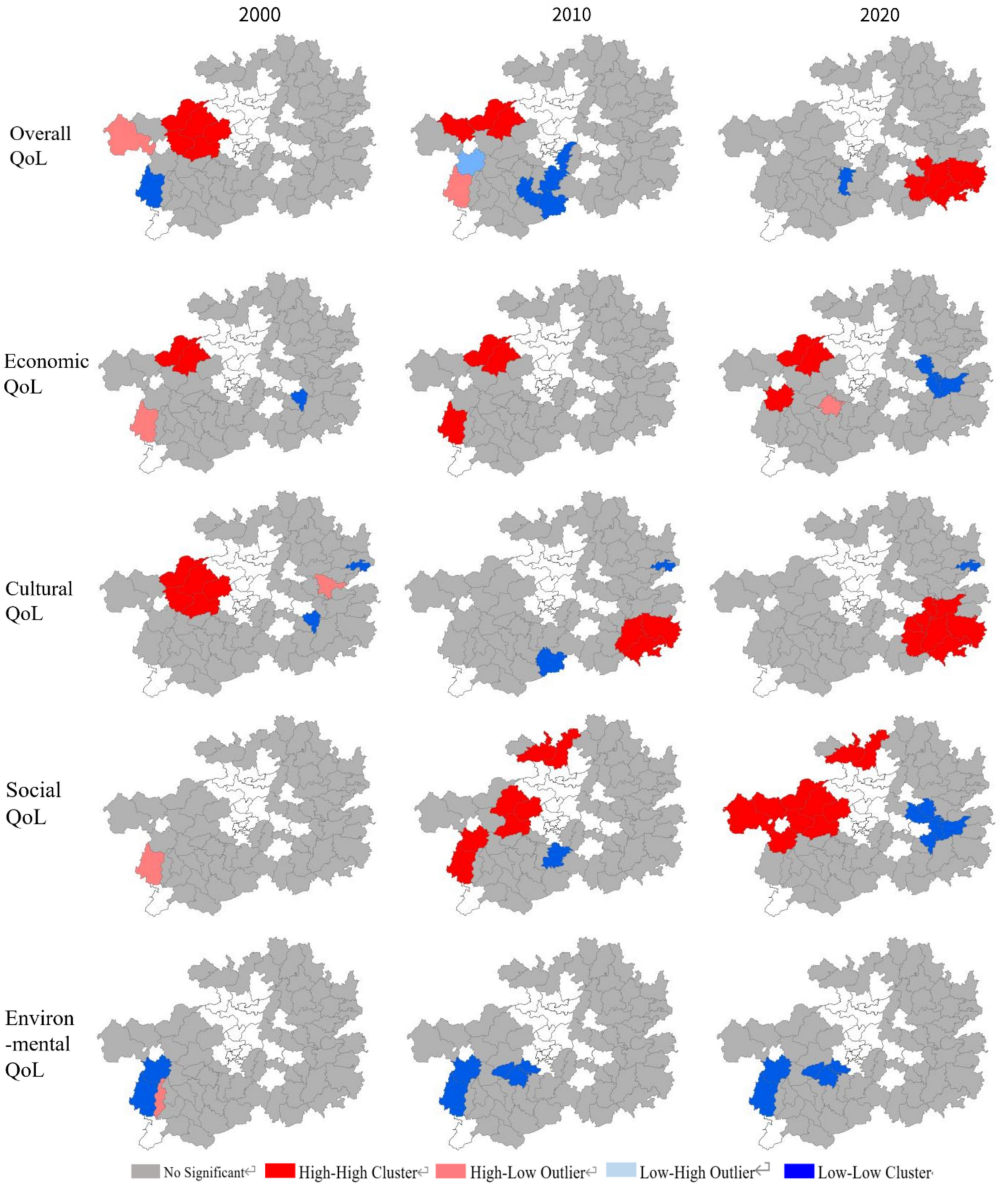
Analysis of the cold and hot spots in the QoL assessments of the impoverished districts and counties of Guizhou Province.

A Local Moran’s I Analysis was performed on the various indexes in the QoL assessment of the impoverished districts and counties of Guizhou Province in 2000, 2010, and 2020, and the corresponding hot spots and cold spots can be presented on maps (see Figure 11).

As far as the overall quality of life of Guizhou’s impoverished districts and counties in the three different years is concerned, the high-value hot spots experienced a shift from the west to the southeast of the province, and the low-value cold spots first spreaded within the southwest and then largely dissolved. Such processes also took place in the spatial clustering evolution of the cultural QoL. Across the three different years, the cultural QoL hotspots among Guizhou’s impoverished districts and counties also relocated from the west to the southeast of the province, while the cold spots gradually vanished.

A different pattern existed in the evolution of the economic QoL of Guizhou’s impoverished districts and counties. The high-value hot spots in this dimension consistently concentrated in the mid-western regions of the province, while the low-value cold spots gradually formed in the mid-eastern regions; The high-value hotspots of social QoL gradually formed in the mid-western and northern regions of Guizhou, while the low-value cold spots gradually disappeared. Finally, the southwestern part of Guizhou Province was consistently a low-value cold spot in terms of environmental QoL in the two decades.

## 5. Discussion and conclusion

### 5.1. Discussion

Guizhou, as the province with the largest number of people overcome poverty in China, has witnessed great changes in the quality of life of its 66 impoverished districts and counties from 2000 to 2020. These changes were also a summary of Guizhou’s unique experience in its development of society, economy, culture and living environment. According to the research outcomes, the development of the overall quality of life in Guizhou was driven by its unique regional ethnic culture and historical culture. However, from the perspective of time distribution, the traction role of cultural quality of life only played an obvious role from 2010 to 2020. From 2000 to 2010, Guizhou mainly promoted the overall quality of life by economic quality of life. In terms of spatial distribution, the overall quality of life in Guizhou was also uneven. The western region had obvious advantages in economic quality of life, while the southeastern region had a strong force in cultural quality of life. The improvement of the overall quality of life in Guizhou also had obvious spatial aggregation characteristics from 2000 to 2020, which was reflected in the disappearance of the spatial aggregation characteristics of the cultural quality of life on the one hand, and the prominence of the spatial aggregation characteristics of the social and economic quality of life on the other hand. The detailed discussion is as follows:

#### 5.1.1. The overall quality of life of Guizhou’s impoverished districts and counties improved over the two decades from 2000 to 2020, while their four specific dimensions of quality of life saw highly varied changes

As shown in the overall QoL assessment results, 87.9% of Guizhou’s impoverished districts and counties experienced a significant improvement in 2000–2020. However, by and large, the economic QoL and the environmental QoL both underwent noticeable declines: 60.6% of all the places studied in the former aspect and 97.0% in the latter. Thus, the improvement in the overall QoL of Guizhou’s impoverished districts and counties were presumably mainly driven by the substantial betterment of their social QoL and cultural QoL.

#### 5.1.2. There existed wide gaps in quality of life between the east and the west among Guizhou’s impoverished districts and counties

In terms of spatial distribution, the improvement of overall QoL in Guizhou’s impoverished districts and counties was rather uneven. While it happened in the west, east and north of the province, the south stagnated throughout the two decades. When it comes to the specific dimensions of quality of life of Guizhou’s impoverished districts and counties, a high economic QoL and social QoL were consistently characteristic of the western part of the province; a high cultural QoL, as a regional feature, shifted from the west to the southeast of the province; and a poor environmental QoL remained typical of western Guizhou. It can be seen that from 2000 to 2020, the social QoL and economic QoL of western Guizhou stayed relatively high. However, the improvement of its cultural QoL lagged that of other regions in the province. Also consistently low was the environmental QoL of this region. In general, the impoverished districts and counties in western Guizhou enjoyed a higher economic QoL and social QoL than similar places within the province, while the southeastern region had a higher cultural QoL and environmental QoL.

#### 5.1.3. Different driving forces for the evolution of quality of life of Guizhou’s impoverished districts and counties in different years

According to the results of the correlation analysis, in 2000, the main driving force behind the improvement of the overall quality of life in Guizhou’s impoverished districts and counties was the economic QoL. In 2010, it was the cultural QoL that played the same role, which was consolidated in 2020. Therefore, the main driving force behind the improvement in the overall QoL of Guizhou’s impoverished districts and counties changed from the economic QoL into the cultural QoL.

#### 5.1.4. The spatial aggregation characteristics of the quality of life of Guizhou’s impoverished districts and counties evolved over time

The global spatial autocorrelation analysis suggests that the overall quality of life of Guizhou Province consistently showed obvious characteristics of spatial aggregation in the two decades between 2000 and 2020. Meanwhile, while its economic QoL and social QoL did not show similar characteristics in 2000, such characteristics were present in these two dimensions in both 2010 and 2020. This indicates that a spatially clustered economic development and social development gradually formed amid the improvement of the overall QoL of Guizhou’s impoverished districts and counties. On the other hand, the opposite process happened in the development of Guizhou’s cultural QoL. While it exhibited obvious characteristics of spatial clustering in 2000, such characteristics were gone in 2020. This suggests that the development of the cultural QoL of Guizhou’s impoverished districts and counties was increasingly a spatially even phenomenon. Due to the uneven distribution of natural resources, the environmental QoL of Guizhou impoverished districts and counties remained spatially clustered.

According to the results of local spatial autocorrelation analysis, the distribution of the cold and hot spots both in the overall QoL and in the four specific dimensions of Guizhou’s impoverished districts and counties showed different characteristics over time. The overall QoL showed different characteristics of spatial aggregation in the three different years. In 2000, the impoverished places with high overall quality of life were clustered in western Guizhou. Such a clustering was less obvious in 2010. And by 2020, it had been relocated to the southeastern region. This development implies that there were interlinked changes in the strategies for improving the quality of life in impoverished districts and counties in various regions. Based on the analysis of the cold and hot spots of the four specific QoL dimensions, it can be inferred that Guizhou’s southeast boasted a clustering of cultural resources, whereas the west enjoyed a linkage between its economic development and its social development. In addition, a low environmental QoL continued to be problematic for the southwest. All in all, the local spatial aggregation characteristics of the four specific dimension of quality of life of Guizhou’s impoverished districts and counties can reveal the potential advantages of those places when trying to improve their quality of life.

### 5.2. Conclusion

By comparing the quality of life of 66 historically impoverished districts and counties of Guizhou province in 2000, 2010 and 2020, the study sums up the distribution characteristics and evolution patterns of the quality of life in those places in the four aspects of economy, culture, society and environment. Both the distribution characteristics and evolution patterns of QoL are visualized through Geographic Information System (GIS). The study introduces spatial autocorrelation analysis into QoL assessment, and examines the change process of quality of life from the angel of spatial agglomeration. This new approach is particularly useful in analyzing QoL transformation and its interregional correlation and can be widely applied in various QoL assessments and related research projects. One notable advantage of this approach is that it can present the similarities and differences in the QoL evolution between regions in a straightforward manner. Thus, it can be used to reveal the potential for coordinated development between regions, which can help authorities formulate development strategies that are customized based on local conditions and needs.

## Data availability statement

The original contributions presented in the study are included in the article/supplementary material, further inquiries can be directed to the corresponding author.

## Author contributions

JY, JD, and HY: conceptualization. JY, HY, and JD: methodology and validation. JY, CC, and HY: software and visualization. JY and HY: formal analysis, writing—original draft preparation, writing—review and editing. JY, HY, and TC: resources and data curation. JD and JY: supervision. All authors have read and agreed to the published version of the manuscript.

## Funding

This research was funded by National Natural Science Foundation of China Regional Science Foundation Project, grant number: 52068006.

## Conflict of interest

The authors declare that the research was conducted in the absence of any commercial or financial relationships that could be construed as a potential conflict of interest.

## Publisher’s note

All claims expressed in this article are solely those of the authors and do not necessarily represent those of their affiliated organizations, or those of the publisher, the editors and the reviewers. Any product that may be evaluated in this article, or claim that may be made by its manufacturer, is not guaranteed or endorsed by the publisher.

## References

[1] Farquhar M. Definitions of quality of life: a taxonomy. J Adv Nurs. (1995) 22:502–8. doi: 10.1046/j.1365-2648.1995.22030502.x 7499618

[2] Costa DSJ Mercieca-Bebber R Rutherford C Tait MA King MT. How is quality of life defined and assessed in published research? Qual Life Res. (2021) 30:2109–21. doi: 10.1007/s11136-021-02826-0, PMID: 33792834

[3] WHOQoL Group. Study protocol for the World Health Organization project to develop a quality of life assessment instrument (WHOQOL). Qual Life Res. (1993) 2:153–9. doi: 10.1007/BF00435734, PMID: 8518769

[4] Douglas O Russell P Scott M. Positive perceptions of green and open space as predictors of neighbourhood quality of life: implications for urban planning across the city region. J Environ Plan Manag. (2019) 62:626–46. doi: 10.1080/09640568.2018.1439573

[5] Zhang S Xiang W. Income gradient in health-related quality of life — the role of social networking time. Int J Equity Health. (2019) 18:44. doi: 10.1186/s12939-019-0942-1, PMID: 30876427 PMC6419834

[6] Terzi F Türkoğlu HD Bölen F Baran PK Salihoğlu T. Residents’ perception of cultural activities as quality of life in Istanbul. Soc Indic Res. (2015) 122:211–34. doi: 10.1007/s11205-014-0688-5

[7] Turkoglu H. Sustainable development and quality of urban life. Proced Soc Behav Sci. (2015) 202:10–4. doi: 10.1016/j.sbspro.2015.08.203

[8] Marans RW. Quality of urban life & environmental sustainability studies: future linkage opportunities. Habitat Int. (2015) 45:47–52. doi: 10.1016/j.habitatint.2014.06.019

[9] Pacione M. Introduction on urban environmental quality and human wellbeing. Landsc Urban Plan. (2003) 65:1–3. doi: 10.1016/S0169-2046(02)00231-1

[10] Huang SCL. A study of outdoor interactional spaces in high-rise housing. Landsc Urban Plan. (2006) 78:193–204. doi: 10.1016/j.landurbplan.2005.07.008

[11] Wang W-M Peng H-H. A fuzzy multi-criteria evaluation framework for Urban sustainable development. Mathematics. (2020) 8:330. doi: 10.3390/math8030330

[12] Shafer CS Lee BK Turner S. A tale of three greenway trails: user perceptions related to quality of life. Landsc Urban Plan. (2000) 49:163–78. doi: 10.1016/S0169-2046(00)00057-8

[13] Smith T Nelischer M Perkins N. Quality of an urban community: a framework for understanding the relationship between quality and physical form. Landsc Urban Plan. (1997) 39:229–41. doi: 10.1016/S0169-2046(97)00055-8

[14] Jiang ZP Yu GL. The impact factors of quality of life among rural-to-Urban migrants. In Proceedings of cross-cultural occupational Health Psychology forum, Wuhan, China (2015). 172–177.

[15] Marans RW. Understanding environmental quality through quality of life studies: the 2001 DAS and its use of subjective and objective indicators. Landsc Urban Plan. (2003) 65:73–83. doi: 10.1016/S0169-2046(02)00239-6

[16] Morais P Camanho AS. Evaluation of performance of European cities with the aim to promote quality of life improvements. Omega. (2011) 39:398–409. doi: 10.1016/j.omega.2010.09.003

[17] Weziak-Białowolska D. Quality of life in cities—empirical evidence in comparative European perspective. Cities. (2016) 58:87–96. doi: 10.1016/j.cities.2016.05.016

[18] Alvarez AL Müller-Eie D. Quality of urban life and its relationship to spatial conditions. Sustainable City XII. (2017) 223:285–96. doi: 10.2495/SC170251

[19] Feneri AM Vagiona D Karanikolas N. Measuring quality of life (QoL) in urban environment: an integrated approach. In Proceedings of the 13th International Conference on Environmental Science and Technology. Athens, Greece 05–07, (2013).

[20] Roches SD Brans KI Lambert MR Ruth Rivkin L Savage AM Schell CJ. Socio-eco-evolutionary dynamics in cities. Evol Appl. (2020) 14:248–67. doi: 10.1111/eva.13065, PMID: 33519968 PMC7819562

[21] Guerrero AM Bennett NJ Wilson KA Carter N Gill D Mills M. Achieving the promise of integration in social-ecological research: a review and prospectus. Ecol Soc. (2018) 23:38. doi: 10.5751/ES-10232-230338

[22] Giannico V Spano G Elia M D’Este M Sanesi G Lafortezza R. Green spaces, quality of life, and citizen perception in European cities. Environ Res. (2021) 196:110922. doi: 10.1016/j.envres.2021.110922 33639147

[23] Min Woong Ki. The social functions of the City culture based on ecological knowledge. Knowledge Lib Arts. (2019) 3:51–82. doi: 10.54698/kl.2019.3.51

[24] Gawlak A Matuszewska M Ptak A. Inclusiveness of Urban space and tools for the assessment of the quality of Urban life—a critical approach. Int J Environ Res Public Health. (2021) 18:4519. doi: 10.3390/ijerph18094519, PMID: 33923193 PMC8123219

[25] Zumaya JQ Motlak JB. The role of environmental indicators in improving the quality of Urban life in the City of Baghdad–a comparative study. IOP Conf Ser: Mater Sci Eng. (2019) 518:022085. doi: 10.1088/1757-899X/518/2/022085

[26] Suldina G Sadyrtdinov R Vladimirova S. Quality of life evaluation for large cities in globalized world. In Proceeding of 18th international scientific conference on globalization and its socio-economic consequences. (2018). 2854–2861.

[27] Glebova I Khabibrahmanova R. Life quality Evaluation in the million-plus population cities of Russia: results of empirical research. Procedia Econom. Finance. (2014) 14:236–42. doi: 10.1016/S2212-5671(14)00707-2

[28] Faka A Kalogeropoulos K Maloutas T Chalkias C. Urban quality of life: spatial modeling and indexing in Athens metropolitan area. Greece ISPRS Int J Geo-Inf. (2021) 10:347. doi: 10.3390/ijgi10050347

[29] Jun BW. Exploring the spatial relationships between environmental equity and Urban quality of life. J Korean Assoc Geogr Inform Stud. (2011) 14:223–35. doi: 10.11108/kagis.2011.14.3.223

[30] Kyung-Joo M Byung-chul L. A study on the analysis of quality of life and policy alternatives for better conditions of Urban life the case of Ulsan Mertopolitan City. Soc Sci Res. (2007) 23:95–125.

[31] Petrovič F Maturkanič P. Urban-rural dichotomy of quality of life. Sustainability. (2022) 14:8658. doi: 10.3390/su14148658

[32] Ma L Liu S Fang F Che X Chen M. Evaluation of urban-rural difference and integration based on quality of life. Sustain Cities Soc. (2020) 54:101877. doi: 10.1016/j.scs.2019.101877

[33] Liu Y Hu Y Sun H Zhou G. Study on residents' quality of life in the context of Urban shrinkage: analysis based on subjective and objective data. J Urban Plan Dev. (2020) 146:3. doi: 10.1061/(ASCE)UP.1943-5444.0000597

[34] Tiran J. Measuring urban quality of life: case study of Ljubljana. Acta Geogr Slov. (2016) 56:58–66. doi: 10.3986/ags.828

[35] Faka A Kalogeropoulos K Maloutas T Chalkias C. Spatial variability and clustering of quality of life at local level: a geographical analysis in Athens. Greece. ISPRS Int. J. Geo-Inf. (2022) 11:276. doi: 10.3390/ijgi11050276

[36] Brown TT Wood JD Griffith DA. Using spatial autocorrelation analysis to guide mixed methods survey sample design decisions. J Mixed Methods Res. (2017) 11:394–414. doi: 10.1177/1558689815621438

[37] Darand M Dostkamyan M Rehmani MIA. Spatial autocorrelation analysis of extreme precipitation in Iran. Russ Meteorol Hydrol. (2017) 42:415–24. doi: 10.3103/S1068373917060073

[38] Zhao X Xianjin H Liu Y. Spatial autocorrelation analysis of Chinese inter-provincial industrial chemical oxygen demand discharge. Int J Environ Res Public Health. (2012) 9:2031–44. doi: 10.3390/ijerph9062031, PMID: 22829788 PMC3397362

[39] Xiao Y Gong P. Removing spatial autocorrelation in urban scaling analysis. Cities. (2022) 124:103600. doi: 10.1016/j.cities.2022.103600

[40] Li S Cheng C Wang X Li Z. Analyzing regional economic disparities based on ESDA in Yangtze River Delta, China. IEEE. (2015) 2015:4530–3. doi: 10.1109/IGARSS.2015.7326835

[41] Ki KY. Analysis of Spatio-temporal changes in Urban Heat Islands using local spatial autocorrelation techniques. J Korean Soc Cadastre. (2021) 37:81–98. doi: 10.22988/ksc.2021.37.1.007

[42] Liu K Wang X Zhang Z. Assessing urban atmospheric environmental efficiency and factors influencing it in China. Environ Sci Pollut Res. (2022) 29:594–608. doi: 10.1007/s11356-021-15692-7, PMID: 34341921

[43] Mou J Zhao X Fan J Yan Z Yan Y Zeng D. Temporal and spatial distribution of air pollution in Shenzhen City during 2014-2016. J Hyg Res. (2018) 47:270–6. 29903282

[44] Shaikh SFEA See SC Richards D Belcher RN Grêt-Regamey A Torres MG. Accounting for spatial autocorrelation is needed to avoid misidentifying trade-offs and bundles among ecosystem services. Ecol Indic. (2021) 129:107992. doi: 10.19813/j.cnki.weishengyanjiu.2018.02.019

[45] Hyun KJ Kim Y-K. Application of spatial autocorrelation for analysis of spatial distribution characteristics of birds observed in Namdaecheon River, Muju-gun, Jeollabuk-do. Korea J Environ Impact Assess. (2013) 22:467–79. doi: 10.14249/eia.2013.22.5.467

[46] Kim H-k Yi MS Bin SD. Regional diffusion of smart city service in South Korea investigated by spatial autocorrelation: focused on safety and urban management. Spat Inf Res. (2017) 25:837–48. doi: 10.1007/s41324-017-0150-2

[47] Balducci F Ferrara A. Using urban environmental policy data to understand the domains of smartness: an analysis of spatial autocorrelation for all the Italian chief towns. Ecol Indic. (2018) 89:386–96. doi: 10.1016/j.ecolind.2017.12.064

[48] Huang HC Chu SH Peng CL Liao TH. The spatial spillover effect of local fiscal expenditure in regional housing market: the case of Taiwan. J Hous and the Built Environ. (2022) 37:1339–65. doi: 10.1007/s10901-021-09895-0

[49] Li Y Derudder B. Dynamics in the polycentric development of Chinese cities, 2001-2016. Urban Geogr. (2022) 43:272–92. doi: 10.1080/02723638.2020.1847938

[50] Moran PAP. The interpretation of statistical maps[J]. J R Stat Soc B. (1948) 10:243–51. doi: 10.1111/j.2517-6161.1948.tb00012.x

[51] Anselin L. Local indicators of spatial association LISA. Geogr Anal. (1995) 27:93–115.

